# Thrombotic complications following bariatric surgery: how medical tourism poses challenges to comprehensive care in obesity medicine

**DOI:** 10.1007/s12328-024-02047-1

**Published:** 2024-10-12

**Authors:** Mandour Omer Mandour, Robert Bakewell, John Ong

**Affiliations:** 1 Department of Hepatology, Cambridge University Hospital, Cambridge, UK; 2 Department of Radiology, Cambridge University Hospital, Cambridge, UK

**Keywords:** Bariatric surgery, Porto-mesenteric thrombosis, Medical tourism

## Abstract

The global prevalence of obesity has more than tripled since 1975. Unfortunately, bariatric surgery waiting lists can last many years therefore many patients seek alternative options such as “medical tourism” by venturing abroad for surgery. We describe two cases of porto-mesenteric venous thrombosis in patients who travelled abroad for bariatric surgery. Upon returning both cases required interventional radiological management, and in the first case, the patient underwent a small bowel resection for bowel ischaemia. Porto-mesenteric complications are significant and have profound lifelong consequences. Therefore, it is imperative that patient education is significantly improved, and more stringent regulations by health authorities are put in place to avoid the growing complications of negative health tourism.

## Introduction

Obesity is defined as ‘’abnormal or excessive fat accumulation that may impair health’’ [1]. According to the World Health Organisation (WHO), the global prevalence of obesity has more than doubled since 1990 with 16% of the adult population being obese in 2022 [1]. Obesity is strongly associated with type 2 diabetes and the metabolic syndrome. Body mass index (BMI) is the most practical method to identify obesity at a population level; however, it is not the most accurate metric because of ethnic variations in muscle mass. A BMI equal to or greater than 30 kg/m^2^ is the cut-off for obesity. In the United Kingdom, a BMI > 35 with comorbidities such as type 2 diabetes and obstructive sleep apnoea, or a BMI > 40, are criteria for bariatric surgery [2]. However, a BMI > 35 regardless of the presence or absence of comorbidities, is a criterion for bariatric surgery in the United States [3]. With the global rise of obesity, the volume of weight loss procedures performed each year has risen exponentially with approximately 600,000 procedures registered in 2021 alone [4].

In the United Kingdom, to access bariatric surgery, patients must undergo a four-tier weight management system. The first tier is delivered by general practitioners and practice nurses in primary care where advice and lifestyle advice are given. The second tier involves intervention in the community, e.g. pharmacotherapy. The third tier involves management by a specialist multi-disciplinary team (MDT) and a specialist weight management programme. Lastly, the fourth tier involves severe and complex bariatric services where intense input from obesity medicine MDTs and bariatric surgery are offered. However, the surgical waiting times are long and could last many years. As a result of this, many patients who can afford medical tourism and prefer to have minimally invasive procedures, often choose to venture privately either in the United Kingdom or neighbouring European countries where costs are cheaper. This is not just limited to the United Kingdom as similar trends are seen across the United States and many countries in Western Europe [5]. However, the fields of bariatric medicine and surgery are still evolving and robust international guidelines for post-interventional care are severely lacking. As a compound effect of poorly audited procedures performed privately or overseas, together with the paucity of international practice guidelines, we frequently witness patients who present with complications of such interventions. Here, we report two such cases where patients developed significant portal vein thrombosis after procedures performed privately and overseas.

### Case 1

**Fig. 1:**
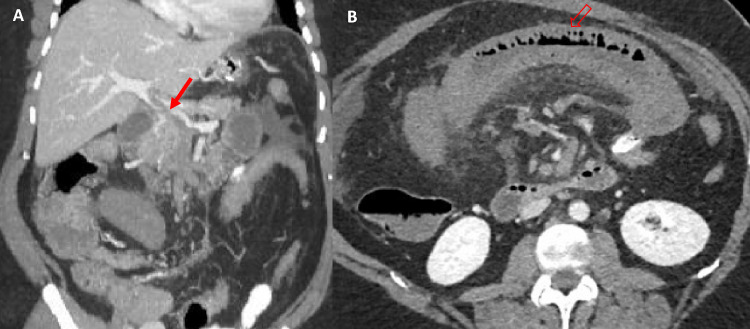
Case 1. **A** Coronal MIP image of a CT examination showing partially occlusive portal and splenic vein thrombus and occlusive superior mesenteric vein thrombus (solid arrow). **B** Axial image from the same CT showing an oedematous and dilated loop of jejunum in keeping with ischaemia (unfilled arrow)

A 46-year-old female with a background of smoking nicotine, previous provoked right lower limb deep vein thrombosis (DVT), and obesity (BMI of 42 kg/m^2^, weight 116 kg) underwent a laparoscopic sleeve gastrectomy in Türkiye and was discharged with a 10-day prescription of subcutaneous prophylactic low molecular weight heparin (LMWH). She was not on any other regular medications. She returned to the United Kingdom 4 days after her operation and presented to her local hospital 2 days later (6 days post-op) with severe right upper quadrant and epigastric pain. Her blood results on presentation are summarized in Table 1. She underwent an urgent computerized tomography (CT) of her abdomen and pelvis which showed a partially occluded portal vein thrombosis (PVT) extending into the superior mesenteric vein (SMV), accompanied by dilated, thickened, and poorly enhancing loops of small bowel suggestive of venous ischemia (Fig. 1).

**Table 1:** Blood results for Case 1

Haemoglobin	146 g/L
WBC	20.7 × 10^9^/L
Platelets	253 × 10^9^/L
INR	1.1 (normal 1–1.15)
Creatinine	120 μmol/L
Sodium	134 mmol/L
Potassium	4.1 mmol/L
Bilirubin	17 μmol/L
Alanine transaminase	28 IU/L
Alkaline phosphatase	125 IU/L
Albumin	35 g/L
C-reactive protein	262 mg/L
Lipase (U/L)	35 IU/L
pH	7.5
Lactate	1.4 mmol/L
Glucose	9.2 mmol/L

She was kept nil by mouth and started on intravenous antibiotics, total parenteral nutrition (TPN), and an unfractionated heparin infusion. A thrombophilia screen including JAK2 mutations was found to be negative. Following a discussion between our HPB surgical team, interventional radiologists (IR) and hepatologists, it was decided to proceed with a transjugular intrahepatic portosystemic shunt (TIPS) with thrombectomy and catheter-directed thrombolysis (CDT) of the PV and SMV to salvage the small bowel. Should this fail, the contingency plan was for an emergency laparotomy and small bowel resection.

**Fig. 2:**
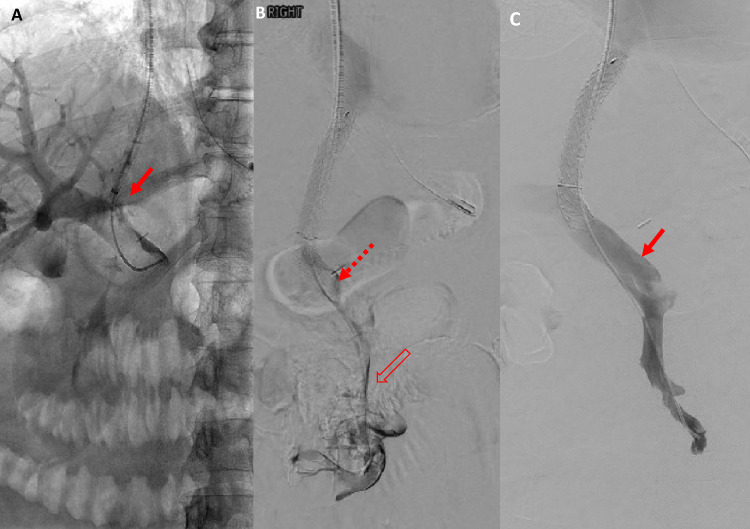
Case 1. **A** Fluoroscopic image from the TIPS procedure (solid arrow). **B** Venography from the same procedure pre-thrombectomy, venoplasty and catheter-directed thrombolysis shows thrombus throughout the superior mesenteric (unfilled arrow) and portal (dashed arrow) veins. Thrombectomy and venoplasty were then performed, and repeated at day 1 and 3, and a thrombolysis catheter left within the portal and superior mesenteric veins until day 5. **C** Repeat venography at day 5 post intervention showing near complete resolution of thrombus (solid arrow)

A TIPS procedure was subsequently performed (Fig. 2) followed by suction thrombectomy and venoplasty to clear the thrombus and improve flow through the SMV and PV. As not all the thrombus could be removed during the initial procedure, a thrombolysis catheter infusing alteplase was placed within the PV and SMV and a systemic heparin infusion continued. Repeat thrombectomy and venoplasty were performed on days 1 and 3, and venography was performed on day 5 with CDT and systemic heparin infusion continuing throughout this period. CT examinations were performed on days 2, 4 and 6 to further assess the patency of the portomesenteric venous system and to monitor ischaemic changes within the bowel. By day 5 there had been a significant reduction in the volume of thrombus within the PV and SMV but unchanged thrombus within small bowel tributaries. CDT was stopped at this point.

**Fig. 3:**
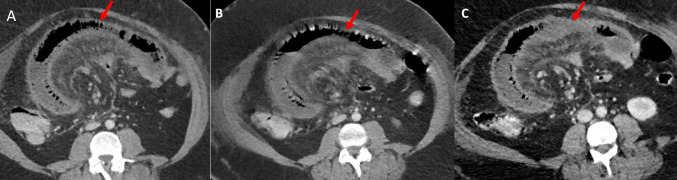
CT examinations at day 2 (**A**), day 4 (**B**), and day 6 (**C**) post presentation showing marked ischaemic change within a loop of jejunum (solid arrow)

Unfortunately, despite these initial promising results, the patient developed worsening abdominal pain over the next 72 h accompanied by fever and rising C-reactive protein levels (maximum value: 368 mg/L) despite systemic anticoagulation and intravenous antibiotics. CT at day 6 post-IR intervention showed continued marked ischaemic change within a loop of jejunum (Fig. 3).

**Fig. 4:**
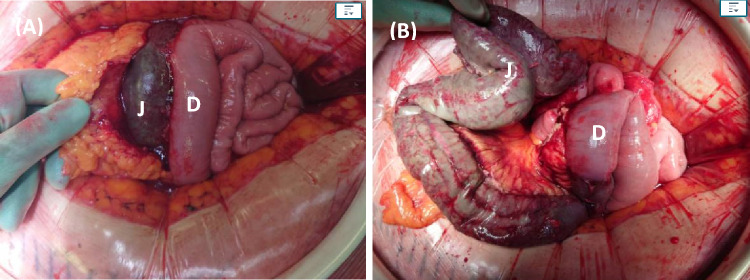
**A** Initial incision revealed a necrotic jejunum (J) and normal duodenum (D). **B** Mobilisation of the small bowel to show the extent of the ischaemia in the jejunum (70 cm). Jejunum resected (70 cm) 15–20 cm of viable jejunum from DJ flexure with rest of jejunum and ileum healthy

A multi-disciplinary decision was made to proceed with surgery and 70 cm of ischaemic jejunum was resected (Fig. 4). The patient made a good recovery with a relook laparotomy after 48 h showing a clean peritoneal cavity with no signs of small bowel ischaemia.

### Case 2:

A 58-year-old female presented to the emergency department with severe epigastric pain and vomiting 2 weeks following a sleeve gastrectomy performed in Türkiye. There was no history of intra or post-operative complications following her surgery. She otherwise had a past medical history of depression; metabolic dysfunction-associated steatotic liver disease (MASLD), and pre-diabetes. There was no history of previous venous thromboembolism (VTE). Her only surgical history of note was an appendicectomy. She had one previous episode of ‘’coffee ground’’ vomiting and passing “black stool”. Her vitals on admission were blood pressure 119/67, heart rate 114, temperature 37 °C, respiratory rate 20, and oxygen saturation 99% on room air. On examination, she appeared clinically dehydrated and had abdominal discomfort. There was evidence of central adiposity with five recent keyhole scars on the abdominal wall that appeared clean and dry. She was tender in the epigastrium and central abdomen with evidence of guarding. Her blood results on presentation in the UK are summarised in Table 2.

**Table 2:** Blood results for Case 2

Haemoglobin	144 g/L
WBC	13 × 10^9^/L
Platelets	145 × 10^9^/L
Mean cell volume	86.6 fL (normal 80–99 fL)
INR	1.2 (normal 1–1.15)
Creatinine	58 μmol/L
Sodium	136 mmol/L
Potassium	3.6 mmol/L
Bilirubin	19 μmol/L
Alanine transaminase	42 IU/L
Alkaline phosphatase	82 IU/L
Albumin	29 g/L
C-reactive protein	128 mg/L
pH	7.3
Lactate	1.18 mmol/L
Glucose	4.2 mmol/L

**Fig. 5:**
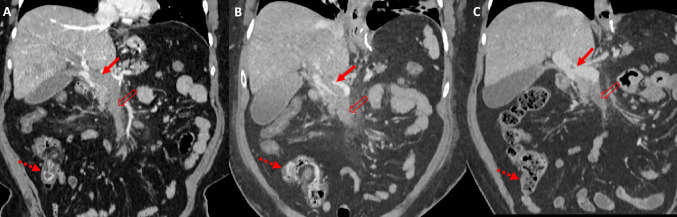
Case 2. Coronal reformats of CT examinations performed at day 0 (**A**), day 3 (**B**), and day 15 (**C**) post admission showing a progressive reduction in thrombus burden within the portal vein (solid arrow) post systemic thrombolytic and anticoagulant therapy. Note unchanged thrombus within the superior mesenteric vein (unfilled arrow), but improvement in the oedema of the caecum and ascending colon (dashed arrow)

Urinalysis did not show any signs of infection or haematuria. She was kept nil by mouth and started on intravenous fluids, antibiotics, and TPN. An urgent CT abdomen was arranged which showed extensive portal and SMV thrombus with no contrast opacification in the main right and left portal veins or SMV. There was also a focal thrombus in the splenic vein and mild mural thickening of the ascending colon (Fig. 5). Otherwise, there was no evidence to suggest developing venous ischaemia of the bowel at the time of scanning. She was discussed with the interventional radiology team who opined TIPS was not feasible because there was no flow in the intrahepatic portal vein. Therefore, she was started on systemic thrombolysis with alteplase (0.05 mg/kg/h–4 mg/h) and unfractionated heparin with a view to reassess in 72 h. A repeat CT the next day showed stable appearances. She received a total of 72 h of thrombolysis and a further CT was performed for re-evaluation. This showed improvement in the volume of thrombus within the main portal vein but continued occlusive SMV thrombosis. The left portal vein and splenic vein thrombi were noted to have resolved. Residual oedema of the right colon was also observed.

Given the improved appearance on CT, systemic thrombolysis was continued for 48 h. Unfortunately, the patient developed significant bleeding from a peripherally inserted central catheter (PICC) site, and thrombolysis had to be stopped. 24 h later she was started on split-dose (twice daily) therapeutic LMWH and her abdominal pain had improved. She was allowed to have oral fluids and slowly built up her oral intake. Following a further week of split-dose LMWH, she improved significantly and her abdominal discomfort had resolved completely. She underwent a repeat CT scan that showed the right portal vein remained completely occluded. However, there was an interval reduction in the volume of thrombus within the intra and extrahepatic main portal vein with a small non-occlusive thrombus in the intrahepatic portal vein. The left portal vein was patent with further re-cannulation of the left lateral inferior branch compared to the previous CT. There was persistent extensive occlusion of the SMV with thrombus and thrombosed collaterals between the IMV and SMV. There was near complete resolution of the oedematous appearing colon with the disappearance of the previously seen free fluid in the pelvis. It was decided she was medically fit for discharge at this point and was discharged on LMWH with repeat imaging arranged at 3 months in view of switching her to oral anticoagulation at that stage.

## Discussion

In an online survey of bariatric surgical members of the international bariatric club (IBC), 64 of 93 responders operated on bariatric tourists with 22% of bariatric surgeons reporting complications [5]. VTE complications were 7.8% and only 12.7% of all patients maintained follow-up until the removal of stitches. More strikingly, only 72.6% recommended follow-up with bariatric teams in the country of origin [5]. In comparison, registry data suggests a VTE complication rate of 0.33–2.99% in patients undergoing bariatric surgery [6, 7]. In our cases, these patients had not been referred to a bariatric team for follow-up and a thromboprophylaxis management plan was not provided. Post-operative surgical follow-up was absent and language barriers were faced.

Daigle et al. [8] showed that of eight specific complications following bariatric surgery which included bleeding, VTE, leak, wound infection, pneumonia, urinary tract infection, myocardial infarction and stroke, VTE had the greatest effect on readmission and mortality [8]. Moreover, according to previous estimates, VTE is the cause of 17% of postoperative deaths following bariatric surgery [9]. In view of this, many bariatric surgery centres provide post-operative VTE prophylaxis following surgery, especially since Porto-mesenteric thrombosis (PMT) following bariatric surgery has previously been reported and has the potential to be organ or life-threatening [10].

Obesity in itself is an independent risk factor for VTE [11]. Hotoleanu et al. [12] showed that obesity was associated with a 6.2-fold increase in risk for VTE with the highest risk for those with class II and III obesity [12]. In the obese state, multiple signalling pathways are activated to stimulate an inflammatory response. Adipose tissue is a known inflammatory mediator and IL-1,IL-6, IL-8 and TNF-α are cytokines that are inducers of the acute phase response that can be released by human adipose cells [13]. It is thought that 15–35% of systemic IL-6 is produced from white adipose tissue [14]. IL-6 works with other interleukins to increase thrombopoietin and stimulate megakaryocytopoiesis leading to an increased platelet count [15]. Furthermore, obesity is associated with elevations in coagulation and von Willebrand factor as well as impaired fibrinolysis [16, 17]. Bureau et al. [18] found that central obesity is associated with portal vein thrombosis (PVT) in patients without liver cirrhosis [18]. Given these factors, patients undergoing bariatric surgery are already at a higher risk for VTE.

PMT, defined as partial or complete occlusion of the portal venous system, may involve intra and extrahepatic veins, extending distally to involve the splenic or mesenteric veins. The Yerdel classification is a commonly used method to stage the extent of PVT involvement and this correlates well to clinical severity. Critically, acute PVTs can lead to intestinal infarction and carry a mortality rate of up to 50% [19]. Its associations with a variety of conditions including intra-abdominal infection, cirrhosis, malignancy and a consequence of abdominal surgery are well established. With the advancement of medicine, other risk factors such as inherited thrombophilias and other causes of acquired prothrombotic states are increasingly recognised as causes of PVT. Over the last several decades, reports of PVT have emerged following laparoscopic surgery that did not involve the portal-venous system [20]. The incidence of PVT following bariatric surgery is thought to be between 0.3 and 1% [21–23].

Surgical sleeve gastrectomy is the most practised method of weight loss surgery and constitutes up to 60% of bariatric procedures worldwide [24, 25]. During this surgery, the gastroepiploic and short gastric vessels are usually dissected [26] along with a significant portion of the stomach which may result in abnormal vascular flow in the remnant tissue [21]. Therefore, this surgical technique likely results in an increased risk of PMT compared to other methods of weight loss surgery [27]. In contrast, the Roux-en-Y gastric by-pass method preserves the main body of the stomach and its natural anatomy, and adjustable gastric bands avoid transection of the stomach and injury to gastric vessels which may explain the markedly lower occurrences of PMT observed with these techniques [27]. Bilio-pancreatic diversion, another method of weight loss surgery, is now infrequently used [21] but there have also been reports of PMT after these procedures [28]. Nonetheless, it is important to consider that the comparatively higher volumes of surgical sleeve gastrectomies may be a confounding factor.

Clinical presentation typically may include abdominal pain, fever, diarrhoea and gastrointestinal bleeding. When thrombosis extends to the mesenteric or splenic veins, patients may present unwell with signs of bowel ischaemia, septic shock and multi-organ failure. The median onset of diagnoses may range from day 1 post-surgery to day 28 [29]. Diagnoses are typically made following cross-sectional abdominal imaging. Chronic PVT may have profound lifelong complications including non-cirrhotic portal hypertension with ascites and varices. It is, therefore, necessary that management not only include lifesaving treatment, but one must also consider how to prevent lifelong complications if possible.

Besides the pathophysiological factors discussed above, other important and common causes of postoperative complications in medical tourism include inconsistent safety standards and discontinuity in patient care [30, 31]. VTEs after sleeve gastrectomy and Roux-en-Y bariatric surgery can be prevented with extended anticoagulation and an enhanced recovery protocol [32]. None of our cases were provided notes of their operation and the recovery process, and only one patient received low-dose anticoagulation. Importantly, flying after recent surgery increases the risks of VTEs because patients are in a “pro-thrombotic” state and are less mobile than usual. As such, the UK Civil Aviation Authority recommends abstinence from flying for 3–4 weeks after major laparoscopic abdominal surgery like a laparoscopic cholecystectomy [33]. However, in these cases, the patients flew back to the UK within 7 days of surgery because they were not advised appropriately. Therefore, better safeguards are needed for patient protection as more patients turn to foreign medical service providers for bariatric surgery.

With regards to anticoagulation, the 2021 International Society on Thrombosis and Haemostasis guidelines do not recommend the use of direct oral anticoagulants (DOACs) for the treatment or prevention of VTE in the first 6–12 months following bariatric surgery however, this was based on studies involving small patient cohorts [34]. In a randomised clinical trial including 272 patients undergoing bariatric surgery, VTE prophylaxis with 10 mg rivaroxaban was found to be efficacious and safe [35]. Interestingly, the American Association for the Study of Liver Diseases (AASLD) recommends treatment of acute PVTs (within 6 months of presentation) with anticoagulation [36].

In conclusion, with the continuing rise of global obesity, the number of patients seeking bariatric surgery will increase in parallel. Unfortunately, without increased access to bariatric surgery in the public sector, longer waiting times will continue and patients may seek alternative options such as ‘’bariatric tourism’’ without fully understanding the risks that entails. Porto-mesenteric complications are significant and have profound lifelong consequences. Therefore, it is imperative that patient education is significantly improved, and more stringent regulations by health authorities are put in place to avoid the growing complications of negative health tourism.
